# Characterization of Anthocyanins and Anthocyanin-Derivatives in Red Wines during Ageing in Custom Oxygenation Oak Wood Barrels

**DOI:** 10.3390/molecules26010064

**Published:** 2020-12-25

**Authors:** Samanta Prat-García, Joana Oliveira, Maria del Alamo-Sanza, Victor de Freitas, Ignacio Nevares, Nuno Mateus

**Affiliations:** 1 UVaMOX Group, Universidad de Valladolid, Avda. Madrid, 50, 34001 Palencia, Spain; samanta.prat@uva.es (S.P.-G.); ignacio.nevares@uva.es (I.N.); 2 REQUIMTE—LAQV, Department of Chemistry and Biochemistry, University of Porto, Rua do Campo Alegre 687, 4169-007 Porto, Portugal; joana.silva.oliveira@gmail.com (J.O.); vfreitas@fc.up.pt (V.d.F.)

**Keywords:** anthocyanin derivatives, barrel, oxygen transfer rate

## Abstract

The ageing of wines in oak barrels is a key stage in the production of high-quality red wines, with the type of oak chosen and the amount of oxygen received by the wine being the determining factors of the process. This work analyses the effect of ageing the same red wine in barrels with different oxygenation rates for one year (OTR), specifically the effect on the evolution of anthocyanins, their derivatives and the appearance of new pigments according to the oxygen dosage in barrels. Results show that wines aged in High-Wood-OTR barrels have a large quantity of monomeric anthocyanins and wine aged in Low-Wood-OTR barrels presents a major intensity of colour. Moreover, using LC-MS analysis, it was possible to detect and identify different families of anthocyanin derivatives, including the tentative identification of two new aldehyde-flavanol-methylpyranoanthocyanin pigments.

## 1. Introduction

Anthocyanins are a family of water-soluble pigments responsible for the colour of red wines, especially young red wines. The concentration of these pigments decreases significantly in a short period of time during red wine maturation, and this is due to the reactivity of anthocyanins with others wine components like tannins, phenolic acids and other small molecules such as pyruvic acid and acetaldehyde [1,2]. The participation of anthocyanins in different reactions yields to the formation of more stable anthocyanin-derived compounds [3]. Different anthocyanin derivatives are found in red wines during ageing, including flavanol–anthocyanin adducts, flavanol–ethyl–anthocyanin adducts, carboxypyranoanthocyanins (Vitisin A), and 4-hydroxy-, 3,4-dihydroxy- and 3-methoxy-4-hydroxy-phenyl-pyranoanthocyanins, as well as polymeric pigments [4,5] that play an important role in sensory, colour and antioxidant properties [6]. It has been observed that adequate oxygenation of red wines favours the formation of new pigments such as Vitisin A) [7,8]. Other studies have shown that oxygen has a great influence on the stability of the new colouring structures [9]. It has also been reported that red wine ageing in contact with wood increases the content of pyruvic acid derivatives [10] and the fraction of polymeric pigments [4,5].

Wine ageing in barrels receives small amounts of oxygen, and this entry of oxygen into the wine changes with time [11]. The oxygen transfer rate of the wood used to build barrels depends, amongst other things, on its species, geographical origin, density and anatomical properties [12]. In the case of French oak wood barrels, the quantity of oxygen passing through the wood can contribute up to 75% of all the oxygen received by the wine, so the type of wood determines the released compounds but also the quantity of oxygen [13].

Different studies have shown the viability of controlling the oxygen that wines receive during their ageing in barrels [13,14], demonstrating the capability of making barrels with different wine oxygenation rates. A recent work by Prat-García et al., 2020 [15], evaluated the oxygen transmission rate (OTR) of barrels made with the Martinez-Martínez et al.’s 2019 method [16]. This method allows staves to be classified by their potential OTR based on their anatomical properties, besides the traditional grain, obtained by image analysis of the wood. The OTR analysis of the barrels built confirmed that the high-OTR barrels dose the aged wines with twice the oxygen as the low-OTR barrels. The aim of this work was to study the evolution of a red wine aged in French oak barrels with different oxygenation rates and the impact of the oxygen on anthocyanins and anthocyanin derivatives and the colour stability of the red wine.

## 2. Results

### 2.1. Oenological Parameters and Color

The oenological parameters of the wines were analyzed at 6 and 12 months of ageing, and the results obtained are presented in Table 1. Some statistically significant differences were observed in the oenological parameters analyzed for the same wine aged in barrels with different oxygen transfer rates. The results obtained show a low coefficient of variation for all the parameters analysed, which shows the homogeneity in the barrels of the same batch.

Overall, after 6 months of ageing, there were no significant differences in the results of the oenological parameters between the HW- and LW-OTR barrel wines, except for the concentration of ethyl acetate, which was found to be higher in LW-OTR barrel wines (*p* = 0.0012). The results of these same parameters after 12 months of ageing did not show significant differences between the batches of wine aged in high- and low-oxygenation barrels. Overall, these results are in agreement with those obtained by Nevares et al., 2009, which studied wines treated with micro-oxygenation together with alternative products of wood with different levels of toasting [17].

The wine colour changed with the time spent in the barrel, as the formation of the new pigments yields to colour stabilization. It was found that the colour intensity (CI) of all wines increased by almost 3 points in the first 6 months of ageing as a result of the formation of new anthocyanin-derived pigments, highlighting the importance of the increase in the blue component of the colour (%620) (Table 1). A contribution to the significant increase observed for all wines after 6 months of ageing can also arise from the presence of anthocyanin-aldehyde-flavanols pigments such as mv-3-glc-ethyl-cat and mv-3-coumglc-ethyl-cat, which have a maximum absorption wavelength around 540 nm and were described in the literature as contributing to the violet hues observed in red wines in the first months of wine maturation [18]. Wine aged in barrels receives in the first three months round 40% of all the oxygen it will receive throughout the year [11], so this period promotes reactions in which oxygen is involved. Furthermore, the interaction with the compounds that the wood releases into the wine can promote the formation of coloured compounds. The increase in CI has been indicated by other authors, since during wine ageing, oxygen determines the formation of anthocyanin-derived pigments and polymerized pigments, which leads to an increase in colour intensity and stability. The condensation processes between anthocyanins and tannins and copigmentation, leading to the formation of new stable pigment compounds that provide a greater intensity of colour are more resistant to degradation and increase the blue tones at the expense of the yellow tones [9,19,20,21]. However, from 6 to 12 months, a slight decrease in colour was observed in all wines, being less important in the wines of the LW-OTR barrels, which dropped 1.36 points in intensity colour as opposed to 1.54 fir the HW-OTR wines or the control wines. It has been observed that LW-OTR barrel wines, after 12 months of ageing, present a slightly higher colour intensity than HW-OTR barrels wines (*p* = 0.0535), possibly due to the formation of pigments by the interaction of wine and wood compounds. This colour intensity decrease is related to the loss of colouring matter and clarification processes with precipitation of phenolic compounds. As to the browning of the wines, reflected in the absorbance at 420 nm, statistically significant differences were observed between the wines of the three types of barrels (*p* = 0.0001) and between the wines of the barrels with controlled oxygenation rate (*p* = 0.0000), with the wines aged in LW-OTR barrels being the ones with slightly lower %420. As anticipated, these results suggest that wines aged in LW-OTR barrels are less oxidized than those aged in HW-OTR.

Moreover, the results obtained for the colour parameters can be correlated to the data obtained in the HPLC analysis, as the decrease in the colour intensity can be due to the decrease observed in the concentration of anthocyanins.

### 2.2. Anthocyanin Content

Table 2 shows the concentration of the 12 anthocyanins detected in the red wines expressed as equivalents of malvidin-3-*O*-glucoside. The mean concentration, standard deviation and coefficient of variation in each control barrels (*n* = 2), LW-OTR barrels (*n* = 4) and HW-OTR barrels (*n* = 4) are represented. The coefficient of variation shows that the concentration of anthocyanins in wines of the same lot of barrels is quite homogeneous and in only five cases exceeds 20% in the analysis (6 and 12 months). The last column indicates whether there are statistically significant differences in the content of each of the compounds in the three types of barrels and between wines from high and low oxygenation barrels (*p* < 0.1).

As described in the literature, the concentration of almost all monoglucoside anthocyanins and the respective acetylated and coumaroylated esters decreased during ageing in barrels, while the concentration of some of their derivatives increased, which is for instance the case of malvidin-3-*O*-glucoside pyruvic acid adduct (A-type Vitisin) [4,5]. It has been observed that after 6 months of ageing, wines from LW-OTR barrels have a higher total content of monomeric anthocyanins than wines from HW-OTR barrels. However, after 1 year of barrel aging, wines from LW-OTR barrels suffer less loss of total content of monomeric anthocyanins than wines from HW-OTR barrels, by 43% and 47% respectively. The anthocyanin that undergoes higher loss is delphinidin-3-*O*-glucoside, decreasing between 55% and 59% in the wines aged for one year in barrels, followed by petunidin-3-*O*-glucoside, with a decrease between 44% and 50%, similar to malvidin-3-*O*-glucoside with losses of 43% to 47%; malvidin-3-*O*-acetylglucoside, with decreases between 36% and 40%; malvidin-3-*O*-(6-*p*-coumaroyl)-glucoside, with decreases between 36% and 37%; peonidin-3-*O*-acetylglucoside, with decreases between 27% and 29%, and finally peonidin-3-*O*-glucoside, with the lowest losses corresponding to 26% and 31% of its concentration in wines from LW and HW-OTR barrels (Table 2). The significantly higher decrease observed for tri-substituted B-ring anthocyanins such as delphinidin, petunidin and malvidin, when compared to di-substituted anthocyanins (peonidin), indicates that tri-substituted anthocyanins are more prone to degradation or have a higher reactivity than di-substituted ones. In fact, the anthocyanin that underwent the highest decrease from 6 months to 12 months was delphinidin-3-*O*-glucoside, a tri-hydroxylated anthocyanin known for its high antioxidant and pro-oxidant effects and hence a reduced stability. Moreover, different malvidin-based derivatives were identified in the studied wines after 6 and 12 months (Table 3), showing the high reactivity of this anthocyanin.

The decrease in anthocyanins concentration has been reported by different authors in red wines aged in oak wood barrels [19,25,26]. Del Alamo et al., 2004 [19], reported about a 30% decrease in anthocyanins monoglucoside in wines aged in barrels, as well as a greater stability of monomeric anthocyanin derivatives and a decrease in colour intensity during ageing [4]. Anthocyanins concentration decreases over time according to various studies, and this includes their chemical transformation into more stable pigments [1]. Mateus and de Freitas, 2001, described that in Port wines, the anthocyanins monoglucoside decreases their concentration by 80–90% during one year of ageing in barrel, while the pyruvic acid derivatives decrease between 155% and 45%, depending on the types of adducts that are formed [20]. Other studies showed the degradation of monomeric anthocyanins as a function of temperature following a first-order reaction [21]. This decrease may be related to other compounds present in wines, such as ellagitannins from oak wood, which may react with anthocyanins as described by Quideau et al., 2005 [25]. These authors described a possible bathochromic effect on tannin-anthocyanin condensation reactions, an effect that explains the evolution of the red colour of the wine to the purple colour typical of barrel ageing. This could indicate that the concentrations of hydrolysable tannins extracted from wood were higher on wines aged on LW-OTR and explain the high colour intensity of these wines when compared to wines aged in HW-OTR barrels [27].

At 6 months of ageing, no statistically significant differences were found in the content of anthocyanins in wines aged in barrels with different oxygenation rates. However, it should be noticed that the anthocyanins concentration in the wine of the HW-OTR barrels is slightly higher than that in the wine aged in LW-OTR barrels, a trend that occurs in all the anthocyanins studied. Specifically, the wine from the HW-OTR barrels presents a higher level of delphinidin-3-*O*-glucoside (*p* = 0.0241), petunidin-3-*O*-glucoside (*p* = 0.0278), peonidin-3-*O*-glucoside (*p* = 0.0651), malvidin-3-*O*-glucoside (*p* = 0.0503), petunidin-3-*O*-acetylglucoside (*p* = 0.0513), peonidin-3-*O*-acetylgluc (*p* = 0.0058) and malvidin-3-*O*-acetylglucoside (*p* = 0.0249) than the wine aged in LW-OTR barrels (Table 2).

As already mentioned, after one year of wine barrel ageing, new compounds were found such as malvidin-3-*O*-glucoside pyruvic acid adduct. This pyruvic acid derivative of malvidin usually appears during alcoholic fermentation, and although different authors have not found a direct relationship between their formation and the amount of oxygen present in the wine [7], it cannot be ruled out that oxygen acts as a catalyst during the ageing period, as it has been widely shown that oxygen is an important colour stabilizer [9]. Malvidin-3-*O*-glucoside pyruvic acid adduct is found in a higher concentration in the wine of control barrels (*p* = 0.035), showing no statistically significant differences between wines from high and low oxygenation barrels.

### 2.3. Anthocyanin Identification by LC-MS

**Figure 1:**
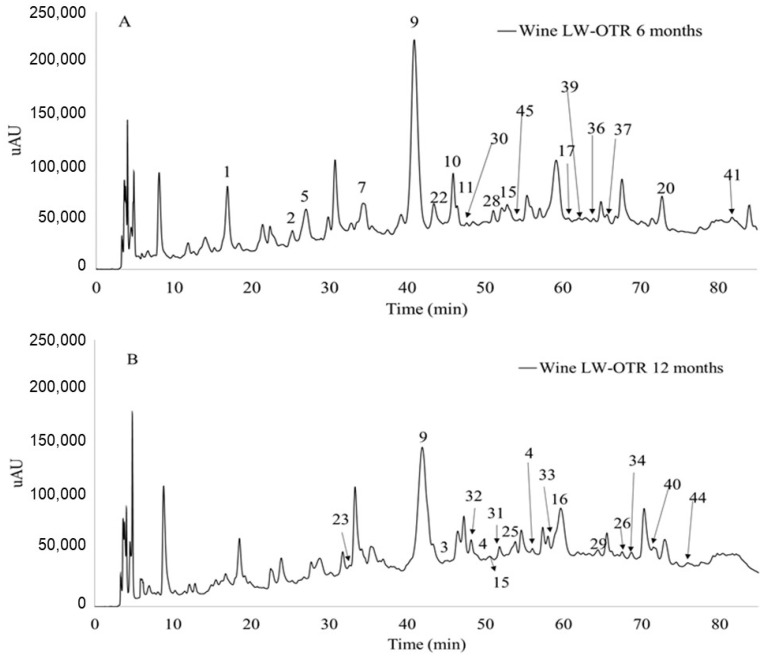
Chromatogram recorded at 520 nm of wine aged in LW-OTR barrels (**A**) aged 6 months (**B**) aged 12 months. The peaks numbers correspond to the compounds identified in Table 3.

Table 3 shows different anthocyanin-derived pigments detected by LC-MS (Figure 1) in the different batches of wine aged for 6 (Figure 1A) and 12 months (Figure 1B) and their classification into different families. Pigment identification was made based on the ion masses and respective fragmentation patterns and comparing with those found in the literature.

The five monoglucoside anthocyanins (dp, cy, pt, pn and mv) and their corresponding acetylated and coumaroylated esters were found in all the red wines aged in different OTR barrel conditions after 6 months. Moreover, the lactate derivative of mv-3-*O*-gluc (14) first described by Alcalde-Eon et al., 2006, was also detected in small amounts after 6 months of wine ageing [10]. In addition, small amounts of two diglucoside anthocyanins (Pt and Dp) (*m*/*z* 641 and 627, respectively) were similarly detected in wines based on their fragmentation patterns (loss of two glucose units) yielding the petunidin and delphinidin aglycones (*m*/*z* 317 and 303, respectively). The diglucoside anthocyanins that are usually present in non—*Vitis vinifera* species have already been identified by LC-MS in *Vitis vinifera* wines and grapes [26].

Oligomeric anthocyanins like dimers (compounds **25** and **27**) and the malvidin-3-*O*-glucoside trimer (compound **24**) were identified by their ion masses and respective fragments in wines aged 12 months. These pigments were already described as occurring in grapes, wines and their by-products [22,28,29]. Moreover, the hydrated form of an ethyl bridge malvidin-3-*O*-glucoside dimer that was firstly described by Atanasova et al., 2002 [28], was also detected in 12-month wines.

Anthocyanin-flavanol adducts, namely malvidin-3-*O*-glucoside-catechin and the gallocatechin derivative, were found to occur in all wines at 6 and 12 months (Control, LW-OTR and HW-OTR). The acetylated malvidin adducts were only found to occur in 12-month aged wines. These pigments are formed from the direct reaction between anthocyanin and flavanol involving the nucleophilic attack of the carbon C-6 or C-8 of a flavanol unit to the electrophilic carbon C-4 of an anthocyanin in the flavylium cation form [4,5].

Concerning pyranoanthocyanins, different families of these pigments were detected in all the wines studied (Table 3). This includes A and B-type vitisins that are formed in wines from the reaction of anthocyanins with pyruvic acid and acetaldehyde, respectively [5]. These anthocyanin-derived pigments present an increased colour stability when compared to their anthocyanin precursors [1]. The concentration of A-type Vitisin (malvidin-3-*O*-glucoside pyruvic acid adduct) was not correlated with the oxygen transfer rate in wines as the concentration of these pigments was not significantly different between wines from control, LW-OTR and HW-OTR barrels (Table 3). On the other hand, in the wine model solution, the presence of oxygen increases the yield of formation of A-type Vitisins [8]. Studies show that the concentration of B-type Vitisin increases during micro-oxygenation and decreases with the end of treatment [29]. The detection of this type of anthocyanin derivatives in the different batches of wine analysed occurs after 6 and 12 months of ageing.

Pyranoanthocyanin-flavanol pigments that were postulated in the literature to be obtained in wines from anthocyanin-alkyl/aryl-flavanol pigments were found to occur in red wines aged12 months. In fact, several anthocyanin-alkyl-flavanol pigments (compounds **45**–**49**) were detected by LC-MS in all wines after 6 and 12 months of ageing in barrel. Pyranoanthocyanin-flavanol compounds contribute to the red/orange colour of wines since their maximum absorption wavelength is around 500 nm.

Pyranoanthocyanin-phenol pigments are formed in wines by the direct reaction between anthocyanins and hydroxycinnamic acids or vinylphenols formed upon microbial decarboxylation of hydroxycinnamic acids [30,31]. These compounds absorb at ~500 nm and also contribute to the red/orange hues observed in wines during ageing.

In addition, in all the wines aged in oak barrels, it was also possible to detect different ion masses (compounds **33**–**41**), namely *m*/*z* 819, 847, 833, 861, between others, presenting the same final fragment at *m*/*z* 531. The first fragmentation pattern in all of them included a sugar moiety that can be esterified with acetic or coumaric acid, indicating that these compounds should correspond to anthocyanin derivatives. On the other hand, the second fragmentation pattern was not the same for all the ion masses detected. Based on the second fragmentation pattern, the compounds were divided into different families. For instance, compounds **33**–**36** that lose a 126 a.m.u. residue (RDA) should present a catechin unit ((+)—catechin or (−)—epicatechin). Based on the ion mass and respective fragments, these pigments should correspond to a flavanol unit linked by the carbon 4 to the carbon 8 of a methylpyranoanthocyanin. Similar pigments have already been described in the literature by Nave et al., 2010 [32]. In this study, the authors hemisynthesized different flavanol-(4–8)-vitisins pigments based on A and B-type vitisins and detected their occurrence in red table wines using mass spectrometry.

**Figure 2:**
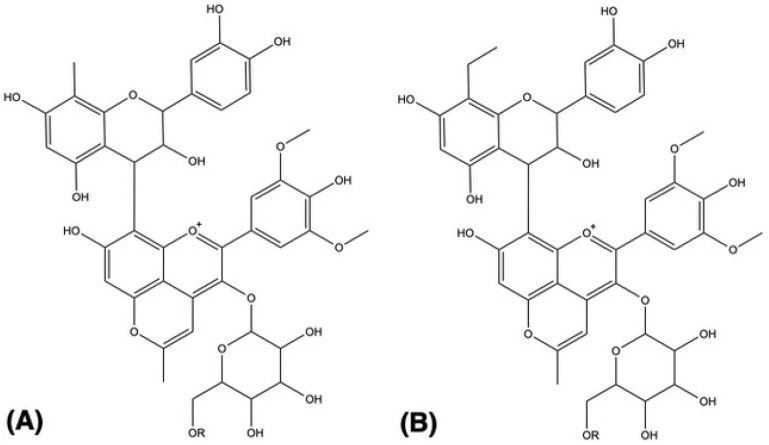
Proposed structure for the (**A**) methyl-catechin-methylpyranoanthocyanins (compounds **37**, **38**) and (**B**) ethyl-catechin-methylpyranoanthocyanin pigments (compounds **39**–**41**). R = H, acetyl or coumaroyl group.

Furthermore, pigments 39–41 present, additionally to the sugar moiety fragment, the loss of a 154 a.m.u. residue. This fragment should correspond to an RDA (126 a.m.u.) typical of a catechin plus an ethyl group (28 a.m.u.) linked to the carbon 6 or 8 of the flavanol moiety. This indicates that these pigments can correspond to an ethyl-flavanol-(4,8)-methylpyranoanthocyanins pigment (Figure 2B). Similarly, pigments 37 and 38 that present a 140 a.m.u. fragment should correspond to an RDA linked to a methyl group, and by this, the two pigments can be identified as methyl-flavanol-(4,8)-methylpyranoanthocyanins pigments (Figure 2A).

As far as the authors know, this is the first time that ethyl and methyl-flavanol-methylpyranoanthocyanin pigments were tentatively identified in red wines.

## 3. Materials and Methods

### 3.1. Oak Wood Barrels

A total of 16 customized oxygenation barrels (Bordeaux-225-L) were made by INTONA S.L. cooperage (Navarra, Spain). For the construction of these barrels, fresh oak staves of *Q. petraea* previously classified by their potential OTR as reported by Martínez-Martínez et al., 2019 [14], were used. The classification of the fresh staves was carried out based on the structural features of the oak staves by means of Artificial Neural Network to predict OTR. The characteristics of each fresh stave were obtained by image analysis of the heads of each stave and based on the potential OTR obtained from the artificial neural network (ANN), and they were classified into three groups: High-OTR, Low-OTR and the rest [16]. Finally, the staves for building 8 barrels with High-OTR and 8 barrels with Low-OTR were selected from the classified groups based on genetic algorithms to obtain a homogeneous group of high oak wood OTR (HW-OTR) barrels and low oak wood (LW-OTR) barrels. In addition, 4 commercial barrels were constructed without any stave classification in the same cooperage with the same but unclassified oak wood, referred to here as control barrels. Half of these barrels (*n* = 4 HW-OTR, *n* = 4 LW-OTR and *n* = 2 control) were analyzed to measure the real OTR according to the method in [11]. The results showed statistically significant differences between the two groups of barrels, with high OTR barrels dosing twice as much oxygen as low OTR barrels. The oxygen input rates in the stationary state were 1.29 and 0.38 hPa/L.day of the HWOTR and LWOTR barrels, respectively. This value reflects the quasi-stationary state of the barrel’s OTR after the initial degassing of the wood and, as described in previous works, is a dynamic value that, with an exponential trend, tends to decrease until day 80–90, after which it can be considered stable [15]. The other half of the barrels built (*n* = 4 HW-OTR, *n* = 4 LW-OTR and *n* = 2 control) were used for the ageing of the same red wine for one year.

### 3.2. Red Wine and Color Parameters

A red wine, from Ribera del Duero region and made from Cabernet Sauvignon grapes of the 2017 harvest, was used. The initial wine presented pH 3.89, alcoholic degree 15% (*v*/*v*), 5.1 total acidity (g/L tartaric acid), 0.48 volatile acidity (g/L acetic acid), 29 mg/L of free SO_2_ and 74 mg/L of total SO_2_, 15.1 colour intensity, % Abs 420 of 38.4, % Abs 520 of 50.1, % Abs 620 of 12.1 and a total polyphenol index of 66 according to OIV, 2003 [33]. The colour analysis was measured with UV/Vis Spectrometer Lambda 25 (Perkin Elmer, Singapore). Each measurement was made in duplicate according to Glories [34].

The wine was barrelled (March of 2018) in 10 barrels (225 L)—4 barrels with High wood-OTR, 4 barrels with Low wood-OTR and 2 control barrels constructed without traceability on the staves, used as control—to age the red wine. The barrels were maintained at 17 °C and with a relative humidity of 70% similar to the common cellar conditions. Wine samples were taken after 6 and 12 months of ageing, after which samples were stored at 18 °C until analysis and the barrels were filled after each sampling. Each sample from each barrel was analyzed in duplicate.

### 3.3. Anthocyanin Quantification

Wine aged in barrels with different OTR were analyzed by HPLC/DAD at 6 and 12 months. The analysis was performed using a Thermo^®^ Scientific Spectra System P4000 pump on a 250 × 4.6 mm i.d. reversed-phase C18 column (Merck^®^, Darmstadt, Germany) thermostatted at 25 °C. The detection was carried out between 200 and 800 nm using a Thermo^®^ Scientific Spectra System UV8000 diode array detector. The volume of sample injected was 20 µL using an autosampler Thermo^®^ Scientific Spectra System AS3000. The solvents were (A) 10% (*v*/*v*) formic acid and (B) 10% (*v*/*v*) formic acid and 30% (*v*/*v*) acetonitrile. The gradient consisted of 20% to 85% of eluent B for 70 min. Identification was carried out using external standards by comparison with retention times and UV/Vis spectrum. For the quantification of anthocyanins, a calibration curve of malvidin-3-*O*-glucoside was used in a concentration range of 75 to 750 mg/L.

### 3.4. Anthocyanin Derivatives Identification

Wine fractionation: 250 mL of each wine sample was fractionated by LiChroprep^®^ RP-18 (40–63 µm, Merck, Germany) using 1 L of acidified aqueous solutions (with 2% HCl) with different percentages of methanol (20%, 40%, 60% and 80%) using a procedure similar to that described in the literature for the fractionation of a grape pomace extract [24]. The obtained fractions were concentrated by rotoevaporation, and the composition of each fraction was determined by HPLC/DAD-MS.

MS analysis: To identify all the anthocyanin-derived pigments present in red wines, all the wine samples and the respective fractions were analyzed by LC/DAD-MS on a Finnigan Surveyor series liquid chromatograph, equipped with a Thermo Finnigan (Merck^®^, Darmstadt) reversed-phase column (150 mm × 4.6 mm, 5 μm, C18) with controlled temperature (25 °C). Solvents were (A) 1% (*v*/*v*) formic acid and (B) 1% (*v*/*v*) formic acid, 30% (*v*/*v*) acetonitrile. The gradient consisted in going from 80% to 15% of A solvent over 70 min, at a flow rate of 0.3 mL/min. The sample injection volume was 20 μL. Double-online detection was done by a photodiode spectrophotometer and mass spectrometry. The mass detector was a Finnigan LCQ DECA XP MAX (Finnigan Corp., San Jose, CA, USA) quadrupole ion trap equipped with atmospheric pressure ionization (API) source, using electrospray ionization (ESI) interface. The vaporizer and the capillary voltages were 5 kV and 4 V, respectively. The capillary temperature was set at 325 °C. Spectra were recorded in the positive ion mode between *m*/*z* 120 and 2000. The mass spectrometer was programmed to do a series of three scans: a full mass, a zoom scan of the most intense ion in the first scan and an MS–MS of the most intense ion using relative collision energies of 30 and 60.

### 3.5. Statistical Analysis

All the analyses of the wine collected from each barrel were carried out in duplicate, and the results are presented as an arithmetic mean ± standard deviation. Wines from eight classified LW-OTR and HW-OTR barrels and two control barrels were analyzed. A statistical analysis of different parameters was evaluated by Statgraphics Centurion XVI (Statistical Graphics Corporation, The Plains, VA, USA) version 16.2.04 for Windows. One-way analysis of variance (ANOVA) at an alpha level of 10% with Fisher post-hoc least significant difference.

## 4. Conclusions

The classification of staves by their OTR to build barrels allows for different contributions of oxygen to the wine, therefore obtaining wines with different anthocyanin and colour characteristics. This study confirmed that the oxygen entry through oak wood influences the colour of red wine during ageing. Some differences in anthocyanins concentration were observed in wines aged in barrels with different OTR.

Moreover, different anthocyanin derivatives were identified through LC-MS analysis, including the detection of A- and B-type vitisins, oligomeric anthocyanins, pyranoanthocyanin-flavanols, pyranoanthocyanin-phenols and anthocyanin-aldehyde-flavanols. Flavanol-methylpyranoanthocyanins and methyl- and ethyl-flavanol-methylpyranoanthocyanin pigments were also tentatively identified for the first time.

## Figures and Tables

**Table 1:** Oenological parameters of the same wine aged in different customized oak wood barrels and wine colour parameters.

		Control Barrels		LW-OTR Barrels		HW-OTR Barrels			
		Mean ± σ	C.V.	Mean ± σ	C.V.	Mean ± σ	C.V.	*p*-Value ^1^	*p*-Value ^2^
Alcoholic degree (% vol)	6 months	15.11 ± 0.01	0.094%	15.08 ± 0.01	0.099%	15.08 ± 0.02	0.14%	0.1586	0.8555
Total acidity (g/L)		5.01 ± 0.02	0.42%	4.95 ± 0.02	0.35%	4.95 ± 0.04	0.73%	0.0983 *	0.8100
Volatil acidity (g/L)		0.67 ± 0.02	3.19%	0.70 ± 0.03	3.92%	0.72 ± 0.01	1.81%	0.0816 *	0.4425
pH		3.86 ± 0.01	0.18%	3.85 ± 0.01	0.21%	3.85 ± 0.00	0.13%	0.6991	0.6202
Acetaldehyde (mg/L)		8.50 ± 0.71	8.32%	10.00 ± 0.82	8.16%	10.00 ± 0.82	8.16%	0.1278	1.00
Ethyl acetate (mg/L)		71.50 ± 2.12	2.97%	70.00 ± 1.83	2.61%	64.50 ± 0.58	0.89%	0.0012 ***	0.001 ***
CI		18.45 ± 0.07	0.39%	18.20 ± 0.40	2.19%	17.94 ± 0.10	0.58%	0.0177 **	0.0991 *
%420		37.38 ± 0.10	0.26%	37.21 ± 0.08	0.22%	37.42 ± 0.05	0.13%	0.0001 ****	0.0000 ****
%520		48.76 ± 0.15	0.31%	48.91 ± 0.10	0.21%	48.76 ± 0.06	0.12%	0.0121 **	0.0024 ***
%620		13.86 ± 0.07	0.51%	13.88 ± 0.11	0.81%	13.82 ± 0.02	0.17%	0.3344	0.1659
Alcoholic degree (% vol)	12 months	15.31 ± 0.02	0.14%	15.25 ± 0.01	0.093%	15.26 ± 0.01	0.085%	0.0087 ***	0.6202
Total acidity (g/L)		4.66 ± 0.07	1.52%	4.66 ± 0.03	0.62%	4.72 ± 0.02	0.46%	0.0840 *	0.0132 **
Volatil acidity (g/L)		0.78 ± 0.04	5.44%	0.76 ± 0.02	1.98%	0.76 ± 0.02	2.40%	0.5109	0.8394
pH		3.84 ± 0.01	0.37%	3.84 ± 0.01	0.22%	3.83 ± 0.00	0%	0.1930	0.0498 **
Acetaldehyde (mg/L)		34.00 ± 2.83	8.31%	34.50 ± 7.37	21.37%	36.25 ± 9.74	26.88%	0.9328	0.7841
Ethyl acetate (mg/L)		60.50 ± 0.71	1.17%	60.00 ± 2.58	4.30%	62.00 ± 1.41	2.28%	0.3815	0.2231
CI		16.60 ± 0.34	2.07%	16.84 ± 0.53	3.15%	16.40 ± 0.25	1.51%	0.1250	0.0535 *
%420		38.92 ± 0.17	0.43%	38.61 ± 0.32	0.82%	38.79 ± 0.08	0.20%	0.0852 *	0.1545
%520		47.53 ± 0.09	0.20%	47.71 ± 0.21	0.43%	47.59 ± 0.04	0.09%	0.1004	0.1134
%620		13.54 ± 0.08	0.56%	13.68 ± 0.12	0.90%	13.63 ± 0.07	0.48%	0.1026	0.3443

^1^ Comparison of wines from 3 types of barrels: control, HW-OTR (high oak wood OTR) and LW-OTR (low oak wood OTR) barrels, ^2^ comparison of wine from HW-OTR and LW-OTR barrels * *p* < 0.1, ** *p* < 0.05, *** *p* < 0.01, **** *p* < 0.001.

**Table 2:** Anthocyanins concentration (mg/L in equivalents of malvidin-3-*O*-glucoside) determined by HPLC/DAD of the red wines at 6 and 12 months ageing at custom barrels.

		Control Barrels		LW-OTR Barrels		HW-OTR Barrels			
		Mean ± σ	C.V.	Mean ± σ	C.V.	Mean ± σ	C.V.	*p*-Value ^1^	*p*-Value ^2^
Dp-3-*O*-gluc	6 months	31.6 ± 1.3	4.2%	28.9 ± 6.7	23.1%	32.0 ± 1.0	3.3%	0.3464	0.213
Pt-3-*O*-gluc		32.5 ± 1.3	4.0%	31.3 ± 2.3	7.3%	31.7 ± 1.5	4.8%	0.5875	0.7057
Pn-3-*O*-gluc		10.1 ± 0.5	4.6%	10.5 ± 2.2	20.5%	9.9 ± 0.4	4.1%	0.6685	0.4210
Mv-3-*O*-gluc		231.9 ± 5.5	2.4%	215.7 ± 34.9	16.2%	230.4 ± 4.7	2.0%	0.3614	0.2573
Dp-3-*O*-acetylgluc		2.1 ±0.1	3.1%	2.1 ± 0.5	25.8%	1.8 ±0.1	5.7%	0.4542	0.3346
Pn 3-*O*-acetylgluc		7.3 ± 0.4	6.0%	6.5 ± 1.5	23.1%	7.1 ± 0.2	3.2%	0.3639	0.3676
Mv-3-*O*-gluc py		nq		nq		nq			
Pt 3-*O*-acetylgluc		nd		nd		nd			
Mv-3-*O*-acetylgluc		74.2 ± 2.2	3.0%	67.8 ± 12.2	17.9%	72.0 ± 2.3	3.2%	0.3838	0.3519
Mv-3-(6-*p*-coumaroyl)-gluc		15.8 ± 0.6	4.0%	15.4 ± 0.7	4.5%	15.5 ±0.7	4.4%	0.5622	0.8947
Total		410.0 ± 12.0		378.2 ± 61		400.4 ± 10.9			
Dp-3-*O*-gluc		13.4 ± 1.2	8.9%	13.3 ± 1.0	7.5%	14.4 ± 0.7	5.1%	0.0657 *	0.0241 **
Pt-3-*O*-gluc		16.8 ± 1.0	6.2%	16.6 ± 1.4	8.2%	17.8 ± 0.4	2.3%	0.068 *	0.0278 **
Pn-3-*O*-gluc	12 months	8.3 ±0.4	5.1%	7.9 ± 0.6	7.4%	8.3 ± 0.2	2.6%	0.1435	0.0651
Mv-3-*O*-gluc		127.1 ± 7.9	6.2%	120.9 ± 12.0	9.9%	130.3 ± 3.2	2.4%	0.1193	0.0503
Dp-3-*O*-acetylgluc		nq		5.6 ± 0.4	7.9%	5.7 ± 0.2	4.3%	0.8440	0.844
Mv-3-*O*-gluc py		7.1 ± 1.7	23.7%	5.4 ± 0.2	3.2%	5.8 ± 1.1	19.1%	0.0351 **	0.3027
Pt 3-*O*-acetylgluc		6.0 ± 0.4	6.0%	5.7 ± 0.4	6.7%	6.0 ± 0.8	2.9%	0.1355	0.0513 *
Pn 3-*O*-acetylgluc		5.2 ± 0.0	0.2%	4.7 ± 0.4	7.6%	5.2 ± 0.1	1.8%	0.0083 ***	0.0058 ***
Mv-3-*O*-acetylgluc		45.5 ± 2.7	6.0%	41.7 ± 4.6	11.0%	46.0 ± 1.3	2.9%	0.0438 **	0.0249 **
Mv-3-(6-*p*-coumaroyl)-gluc		10.3 ± 0.8	7.9%	9.7 ±1.0	10.4%	10.0 ± 0.5	5.5%	0.4914	0.5684
Total		239.7 ± 16.1		231.5 ± 22.4		249.5 ± 8.5			

^1^ Comparison of wines from 3 types of barrels: control, HW-OTR and LW-OTR barrels, ^2^ comparison of wine from HW-OTR and LW-OTR barrels. * *p* < 0.1, ** *p* < 0.05, *** *p* < 0.01, nq (non quantified), nd (non detected).

**Table 3:** Anthocyanins and their derivatives identified in red wines aged in barrels with different OTR by LC/DAD-MS (control (C) (*N* = 2), LW-OTR (L)(*N* = 4) and HW-OTR (H) (*n* = 4) at 6 and 12 months of ageing.

Compound	Rt (min)	[M^+^]	[M^2^]	[M^3^]	Identity	6 Months	12 Months
Anthocyanin-flavanol pigments							
**1**	16.76	797	635	467/373	mv-3-*O*-gluc-gallocatechin	C, L, H	C, L, H
**2**	27.26	781	619	467/373	mv-3-*O*-gluc-cat [5]	C, L, H	C, L, H
**3**	45.04	823	619	467/373	mv-3-*O*-acetylgluc-(+)-catechin [5]	-	C, L, H
**4**	48.93	823	619	467/373	mv-3-*O*-acetylgluc-(−)-(epi)catechin	-	C, L, H
**5**	55.67	927	619	467/373	mv-3-*O*-coumaroylgluc-catechin	C, L, H	-
Monoglucoside anthocyanins							
**6**	28.53	465	303		dp-3-*O*-gluc	C, L, H	C, L, H
**7**	31.46	449	287		cy-3-*O*-gluc	C, L, H	-
**8**	36.02	479	317		pt-3-*O*-gluc	C, L, H	C, L, H
**9**	40.5	463	301		pn-3-*O*-gluc	C, L, H	C, L, H
**10**	41.74	493	331		mv-3-*O*-gluc	C, L, H	C, L, H
**11**	46.34	507	303		dp-3-*O*-acetylgluc	C, L, H	C, L, H
**12**	47.05	511	349		mv-3-*O*-gluc chalcone form [4]	C, L, H	C, L, H
**13**	50.82	491	287		cy-3-*O*-acetylgluc	C, L, H	-
**14**	51.34	565	331		mv-3-*O*-lactgluc [10]	C, L, H	-
**15**	53.26	521	317		pt-3-*O*-acetylgluc	C, L, H	C, L, H
**16**	53.94	553	349		mv-3-*O*-acetgluc chalcone form [5]	C, L, H	C, L, H
**17**	59.65	535	331		mv-3-*O*-acetylgluc	C, L, H	C, L, H
**18**	61.12	655	331		mv-3-*O*-cafgluc [5]	C, H	-
**19**	62.5	611	303		dp-3-*O*-coumgluc	C, L, H	C, L, H
**20**	68.2	625	317		pt-3-*O*-coumgluc	C	C, H
**21**	68.57	639	331		mv-3-*O*-coumgluc [5]	C, L, H	C, L, H
Diglucoside anthocyanins							
**22**	28.78	641	317		pt-3,5-*O*-digluc [10]	C,H	-
**23**	44.81	627	465	303	dp-3,5-*O*-digluc [5]	C,H,L	C
Oligomeric anthocyanins							
**24**	32.3	1477	1315	1153	mv-3-*O*-gluc trimer [18]	-	C, L, H
**25**	49.2	1027	865	661	mv-3-*O*-gluc-mv-*O*-acetgluc dimer [18]	-	C
**26**	52.49	1029	849/ 357	687/342/ 295	mv-3-gluc-ethyl-mv-3-gluc hydrated form [5]	-	C, L, H
**27**	69.12	1131	823	661	mv-3-*O*-coumgluc-mv-3-*O*-gluc dimer [10]	-	C, L, H
A-type vitisins							
**28**	47.3	561	399		A-type vitisin [5]	C, L, H	C, L, H
**29**	51.68	603	399		carboxypyranomv-3-*O*-acetylgluc	C, L, H	C, L, H
**30**	64.58	707	399		carboxypyranomv-3-*O*-coumaroylgluc [20]	H	C, L, H
B-type vitisins							
**31**	48.21	517	355		B-type vitisin [22,23]	C, L, H	C, L, H
**32**	53.74	559	355		pyranomv-3-*O*-acet-gluc	-	C, L, H
Flavanol-methylpyranoanthocyanins							
**33**	48.6	819	657	531	(+)-cat-methylpyranomv-3-*O*-gluc [5]	-	C, L, H
**34**	57.77	819	657	531	(−)-epicat-methylpyranomv-3-*O*-gluc	-	C, L, H
**35**	67.26	861	657	531	cat-methylpyranomv-3-*O*-acetylgluc	C, H	C, L, H
**36**	75.34	965	657	531	cat-methylpyranomv-3-*O*-coumgluc	C	-
Methyl-flavanol-methylpyranoanthocyanins							
**37**	66.1	833	671	531	methyl-cat-methylpyranomv-3-gluc [5]	C, L, H	C, L, H
**38**	74.48	875	671	531	methyl-cat-methylpyranomv-3-*O*-acetgluc [5]	C, L, H	H, L
Ethyl-flavanol-methylpyranoanthocyanins							
**39**	61.63	847	685	531	ethyl-cat-methylpyranomv-3-*O*-gluc [6]	C, L, H	C, L, H
**40**	71.38	847	685	531	ethyl-epicat-methylpyranomv-3-*O*-gluc	-	C, L, H
**41**	82	993	685	531	ethyl-cat-methylpyranomv-3-*O*-cumgluc	C, L, H	-
Pyranoanthocyanins-flavanols							
**42**	50.0	805	643	491	pyranomv-3-*O*-gluc-cat [24]	C, H	-
**43**	68.64	805	643	491	pyranomv-3-*O*-gluc-epicat	C, H	-
**44**	76.36	951	643	491	pyranomv-3-*O*-coumgluc-cat	H, L	C, L, H
Anthocyanin-aldehyde-flavanols							
**45**	55.92	809	357		mv-3-*O*-gluc-ethyl-cat [5]	C, L, H	C, L, H
**46**	56.6	985	823	661	mv-3-*O*-cafglc-propyl-cat	C, L, H	C, L, H
**47**	64.97	851	357		mv-3-*O*-acetylgluc-ethyl-cat	C	-
**48**	70.79	955	357		mv-3-*O*-coumglc-ethyl-cat	-	C, L
**49**	80.76	955	357		mv-3-*O*-coumgluc-ethyl-(epi)cat	-	C, L, H
Pyranoanthocyanins-phenol							
**50**	78.23	609	447		pyranomv-3-*O*-gluc-phenol [19,20]	-	C, L, H
**51**	81.96	651	447		pyranomv-3-*O*-acetgluc-phenol [19,20]	-	C, L, H
